# Psychological safety in health professions education: insights and strategies from a global community of practice

**DOI:** 10.3389/fmed.2024.1508992

**Published:** 2025-01-24

**Authors:** Chaoyan Dong, Lisa Altshuler, Nobutaro Ban, Lee Yuen Wong, Fatima Elbasri Abuelgasim Mohammed, Chao Tian Tang, Elizabeth Kachur

**Affiliations:** ^1^Sengkang General Hospital, Education Office, Singapore, Singapore; ^2^New York University School of Medicine, New York, NY, United States; ^3^Aichi Medical University School of Medicine, Nagoya, Japan; ^4^Orthopaedic Surgery Department, Khoo Teck Puat Hospital, Singapore, Singapore; ^5^Faculty of Medicine, University of Khartoum, Khartoum, Sudan; ^6^Department of Psychiatry, Sengkang General Hospital, Singapore, Singapore; ^7^Medical Education Development, Global Consulting, New York, NY, United States

**Keywords:** health professions education, psychological safety, interprofessional education, simulation-based medical education, team-based learning

## Abstract

Psychological safety is the belief that one will not be punished or humiliated for speaking up, sharing ideas, raising concerns, or making mistakes. There are various threats to psychological safety in health professions education (HPE). This commentary applies Clark’s model of psychological safety (Inclusion Safety, Learner Safety, Contributor Safety, Challenger Safety) to five different HPE settings (classroom instructions, clinical training, simulation-based training, online instructions, interprofessional education). Setting-specific threats and strategies for enhancing psychological safety are discussed.

## Introduction

Psychological safety is the belief that one will not be punished or humiliated for speaking up, sharing ideas, asking questions, raising concerns, or making mistakes (1, 2). It is the foundation for a growth mindset (3). Without feeling safe, individuals will avoid taking risks, some of which are necessary for optimal performance. Psychological safety was first studied in the field of organizational behavior focusing on risk-taking behavior in interpersonal relationships and business outcomes (4). The concept made its way into medicine and medical education through the following developments: competency-based medical education (5), patient and workforce safety (6), interprofessional education and team-based care (7), simulation-based training (8), and the recent focus on diversity, equity and inclusion (DEI) in healthcare (9). The Accreditation Council for Graduate Medical Education (ACGME) added psychological safety as one of the core principles for all residency training programs (10).

Psychological safety is important in health professions education (HPE), due to its growing emphasis on diversity, equity and inclusion (DEI) (11). Addressing the DEI matters helps to improve healthcare outcomes (12), addressing systemic issues embedded in healthcare (13), and increasing diversity in faculty and students (14). Empowering educators is crucial for effective implementation of DEI practice (15). To empower educators effectively, addressing and mitigating threats to psychological safety while adopting robust strategies to nurture and maintain it among educators and learners is necessary. This was shown in discussions among our team and participants across 15 workshops at international and regional conferences over the past 5 years.

We advocate for the universal implementation of the principles of psychological safety in health professions education (HPE) by applying Clarke’s model. Compared to other models, this framework is versatile and can be easily adapted to various educational settings. In contrast, other models, such as TeamSTEPPS (16), caters to clinical settings, focusing on improving patient safety by enhancing communication and teamwork skills among healthcare professionals. The Just Culture Model (17) is to create an environment in which everyone feels safe reporting near misses and mistakes, promoting patient safety.

Clark’s 4-stage model describes how a person acquires and embraces psychological safety in teams or organizations (18). “Inclusion Safety” refers to feeling accepted and included as the person you are, irrespective of clinical roles and backgrounds. “Learner Safety” is established if someone feels comfortable asking questions, exploring new topics and learning from mistakes without compromising patient safety. “Contributor Safety” refers to feeling empowered to share one’s knowledge and skills and thus add value to a group or task. “Challenger Safety” signifies readiness to speak up and challenge the status quo without fear of retribution within reasonable boundaries. While challenger safety may appear less relevant to the initial phases of medical training, it is critical for programs that promote innovation, advocacy, allyship and leadership skills.

Psychological safety is context-specific and influenced by cultural factors. What feels safe in one setting may be perceived as threatening in another. Using Clark’s model, we illustrate how individuals navigate transitions between phases as they move across different HPE settings and cultures (19). For example, an individual may experience the “Challenger Safety” in one place but excluded or hesitant to ask questions in another. This highlights the dynamic nature of psychological safety in HPE (20).

## Process of developing these insights and strategies

This document is the culmination of an iterative process spanning 5 years, during which we facilitated 15 workshops focused on psychological safety in Health Professions Education (HPE). Our team includes members from Africa, Asia, and North America, comprising a medical student, three physicians, three medical educationalists, working across the broad spectrum of HPE. These workshops were held at the meetings of international, regional and local conferences, including the Association for Medical Education in Europe (AMEE), the Ottawa Conference, the International Meeting of Simulation in Healthcare (IMSH), the Australian & New Zealand Association for Health Professional Education (ANZAHPE), and SingHealth Duke-NUS Education Conference (21–24). Before these workshops, our team conducted extensive literature reviews and drew on our collective experiences in HPE to design the content. During the workshop, we engaged participants with interactive presentations and led case discussions that explored psychological safety across various HPE contexts. Following each workshop, we compiled and analyzed the key discussion points and integrated our reflections with insights from the literature. This iterative process helped to enrich each subsequent workshop. This article distills our reflections and insights from these workshops, outlining the strategies for fostering psychological safety in diverse HPE settings.

## Strategies to enhance psychological safety in five HPE settings

Table 1 summarizes risks and strategies to enhance psychological safety in five HPE settings: classroom instructions, clinical training, simulation-based education, online teaching, and interprofessional education. We choose different educational methods for each setting to highlight how psychological safety can be achieved.

**Table 1:** Threats and strategies to enhance psychological safety in health professions education using the Clark’s psychological safety stages model.

**Educational Settings**	**Typical Threats to Psychological Safety for Learners**	**Strategies to Enhance Psychological Safety**			
		**Inclusion Safety** (feeling accepted and included)	**Learner Safety** (feeling safe to ask questions and make mistakes)	**Contributor Safety** (feeling safe to use one’s skills and abilities to contribute to the group or task)	**Challenger Safety** (feeling safe to challenge the status quo without fear of retribution)
Classroom Instructions	Complex and overwhelming amounts of foundational knowledge and skills High-stake exams Competition among learners from diverse backgrounds	Create educational activities (e.g., group assignments) that reduce isolation Organize longitudinal groups that encourage relationship development Emphasize collaboration over competition	Encourage and reward question asking Faculty acknowledge limitations and demonstrate willingness to learn Arrange for frequent multi-source feedback	Plan activities that engage all learners equally Welcome all contributions even if they require some corrections	Create debates to signal that expressing alternative opinions is acceptable React to opposing views with curiosity, not dismissal Role model creativity and innovation to encourage it in learners
Clinical Training	Risks of making mistakes and harming patients Interprofessional care team communications and dynamics Emotionally challenging experiences (e.g., patient suffering/death) Overwhelming workload	Longitudinal tracks with consistent preceptors and teams to enhance relationship development Include learners in professional as well as social activities whenever possible	Offer a range of assignments to address diverse learning needs and interests Plan for routine debriefings and reflection activities Create mentoring opportunities	Orient learners to local protocols and cultures upfront to allow for easy integration Assign training-level appropriate roles that can result in valuable contributions to the healthcare team	Provide multiple pathways (including anonymous routes) for reporting unsafe conditions Role model flexibility and effective change management
Simulation-based Training	Pressure to perform well under close observation Complex and intimidating high-fidelity simulators SPs who can make tasks more difficult if their psychological safety is not attended to SPs and faculty who could make biased judgments and provide harsh feedback	Design training-level appropriate culturally diverse scenarios Beware of stereotyping risks due to the use of specific mannequins (e.g., skin tone) and SPs (e.g., age)	Orient learners to goals and objectives (e.g., formative or summative) Reiterate the “Basic Assumptions” to emphasize learners’ intentions to do their best and learn Allow for time-outs by all participants (including SPs)	Assign appropriate roles and responsibilities Prep learners with pre- simulation assignments (e.g., readings, workshops) Require contributions by all learners during debriefing (e.g, round-robin format) Publicly acknowledge and appreciate all contributions promptly	Partner with students to design the simulation Explore alternative strategies during debriefings
Online Instructions	Technical breakdowns that interfere with the learning experience Difficulties reading the reactions of others which results in broken feedback loops Limited access to immediate help from educators and peers	Publicize backup plans that include alternate communication channels and access to technical support Promptly attend to connectivity problems of individual learners which signals to everyone that no one is expendable If possible request turning videos on to facilitate everyone’s “authentic presence”	Use active learning strategies to optimize comprehension (e.g., breakout rooms, polls) Regularly check-in with learners to gauge understanding (e.g., pop quizzes)	Orient learners to timing and contribution expectations upfront Offer multiple channels for learners to contribute Call on individual learners by name Use small group activities in a breakout room to enhance participation	Include panels or debates to showcase different perspectives Ask probing questions to illustrate the value of alternative viewpoints
Inter-professional Education	Institutional hierarchies can threaten psychological safety, especially for those who are on the bottom of the power structure Teams are dynamic; changes can destabilize the social unit Interprofessional stereotypes and biases can affect group cohesion Unclear roles and responsibilities introduce uncertainties	Craft goals and objectives that highlight the unique contributions of all participating professions Assemble a multi-professional faculty team to demonstrate interprofessional collaboration Allocate adequate time for introductions	Introduce activities which allow trainees from different professions to learn from each other Emphasize common goals	Design learning activities in which each participant is assigned a profession-appropriate role Stress the importance of open communications	Rotate leadership responsibilities to examine shifts in team dynamics Practice “speaking up” by using TeamSTEPPS or similar communication frameworks

### Classroom instruction

In pre-clerkship programs, students must acquire a vast amount of complex foundational knowledge, and are pressured to pass high-stakes exams before starting clinical rotations. Team-based learning (TBL) is a widely used education approach (25). It provides excellent opportunities to promote psychological safety. TBL requires creating a space where students feel comfortable taking intellectual risks and expressing themselves without fear of embarrassment or retribution. Below are some examples of how this can be accomplished in TBL.

Pre-class: Instructors set the stage by communicating to students that TBL focuses on learning with and from peers. Providing students with learning materials upfront equips them with the foundation knowledge needed to engage in informed applications and meaningful discussions.

Readiness assurance tests: Before starting a class, students complete the iRATs (Individual Readiness Assessment Tests) and tRATs (Team Readiness Assessment Tests) to identify learning gaps. The scores for these formative assessments are not counted toward the final grades, which reduces students’ stress levels.

Group assignments: By maintaining the same small groups over a long period, relationships and a level of comfort can develop. Role distributions ensure that all students participate and have an equal chance to be heard. Reinforcing that it is “okay to make mistakes” and establishing confidentiality rules can further enhance psychological safety.

Peer evaluation and feedback: This teaches students to provide constructive feedback to each other in a non-threatening way. Requiring everyone to reflect on team dynamics fosters communication and collaboration.

Educator role: Faculty serve as facilitators and role models. They need to encourage students to ask questions and continuously emphasize that making errors is a valuable part of learning. By regularly checking in with students, they can gage learners’ comfort levels and take action to enhance psychological safety if necessary.

### Clinical training

Learners are expected to apply foundational knowledge, acquire further clinical skills, and navigate interpersonal and interprofessional dynamics while maintaining patient safety. With such complex demands on learners, psychological safety is crucial for optimal training in clinical settings.

Longitudinal Integrated Clerkships (LICs) allow students to participate in patient care over an extended period. Thus they can build and deepen relationships with patients and the clinical care teams (26). Different from short-term rotations, this continuous same-setting immersion facilitates the development of psychological safety by helping students become more comfortable admitting uncertainties and asking questions to optimize learning. Here are some examples of how LIC education models can cultivate psychological safety:

Orientation: Students are briefed about expectations, roles, and the importance of psychological safety in clinical learning and patient care. They also receive guidance on program structures, local protocols and cultures (e.g., how to handle challenging situations with patients and other team members).

Longitudinal preceptorship: Attachment to an individual supervisor or a small group of faculty allows regular check-ins to address learning progress and concerns. Supervisors will also feel more comfortable sharing their own experiences, such as learning from mistakes. Such openness can signal to learners that acknowledging one’s vulnerabilities is okay. Faculty development can help preceptors develop skills to provide supportive and non-punitive feedback that is directed toward improvement.

Team integration: Healthcare teams are encouraged to embed students in rounds, case discussions, and relevant decision-making processes. This builds trust and rapport on both sides and helps create a safe learning and work environment (27). Inclusion in social events (e.g., holiday parties) will further enhance a sense of belonging.

Curriculum structure: The longitudinal nature of the program provides many opportunities to build in routine debriefing and reflection activities. This allows students to discuss their learning experience. A reporting system that includes anonymous options to address problematic situations provides an effective recourse to psychologically unsafe conditions.

### Simulation-based training

Simulation has been widely used in healthcare education due to its resemblance to clinical practice and patient safety concerns (28). Simulation centers bring together educators, learners, standardized patients/participants (SPs), technicians, and administrators. Typically, they also include task trainers and high-fidelity simulators that can be intimidating. Even when programs are labeled as “formative,” simulation-based training puts one’s competencies under a microscope, subject to analysis and assessment. Performance anxiety is common, not just for learners, but also for SPs who are enacting their specific roles, and for faculty observers, who must deliver accurate ratings and effective feedback. The simulation community has developed various strategies to combat built-in threats as illustrated below.

- Pre-briefing: It is a standard of best practice for educators to provide a robust orientation and to set the tone for an upcoming simulation event (29). This includes defining goals and objectives, informing everyone involved about the agenda, providing set-up information, and laying down ground rules. Reciting the “Basic Assumptions” (30) highlights the best intentions of all participants to perform well and learn, and creates a safer environment upfront. Additional statements about confidentiality can reduce anxiety further. If the simulation is used formatively, some programs add pre-event workshops or readings to strengthen skills in advance and thus build more confidence going into the simulation activity.
- During the simulation: Implementing simulation-based training requires a multitude of individuals to work together. Sometimes, medical institution hierarchies extend to simulation centers, resulting in disrespect for some team members. SPs are most at risk of being marginalized, which can threaten their sense of safety and will influence case portrayal, evaluation and feedback. The Association of SP Educators (ASPE) created the Standards of Best Practice (31) which highlights psychological safety as an essential part of SP work conditions. While learners are at the receiver’s end of simulation training, there is an increased call to incorporate them in program development. This allows their perspectives and concerns to be fully considered, and it can enhance psychological safety for all.
- Debriefing: Post-simulation debriefing fosters reflection, learning, and growth. Psychological safety is crucial, as learners often feel vulnerable during this phase. Debriefing frameworks (32–34) ensure constructive discussions, focusing on successes and areas for improvement. In group settings, facilitators play a key role in managing dynamics, reinforcing ground rules, and ensuring equal participation to maintain safety. Quiet participants can lower group safety, so their engagement is essential. Effective programs also debrief SPs, addressing the psychological impact of switching in and out of challenging roles, in line with ASPE’s Standards of Best Practice (31). Support SPs’ well-being promotes their sustained engagement.

### Online instructions

Psychological safety can be undermined by concerns about technical system failures, challenges in interpreting others’ reactions, and difficulties accessing support from educators and peers. Ignoring these factors may compromise learning outcomes and disrupt the feedback loop educators rely on to refine their teaching. The post-pandemic shift to technology-mediated learning led to many publications on optimizing this approach (35). Psychological safety is especially important for synchronous interactions between learners and educators or among learners themselves.

- Initiating a positive learning environment: To address faculty and learner anxieties about fast changing technologies and potential breakdowns, back-up plans, such as alternative communication strategies, are established (36). Demonstrating enthusiasm for online teaching sets a more positive tone than expressing reluctance toward the modality. In the post-pandemic era, online learning has transitioned from a necessity to a choice, fostering a more favorable attitude. Learners, often well-versed in technology, can be involved in problem-solving, promoting respect, collaboration, and inclusion within the learning environment.
- Fostering learners’ presence in the virtual space: As with in-person educational session, educators need to be explicit about the learning objectives, time allocations, individual and group activities. Sticking to these structures will confirm predictability that positively impacts psychological safety. Rules of conduct are reviewed upfront. Conventions about keeping video and microphone on or off can differ by culture, institution, bandwidth, and physical environmental factors (37). However, seeing and hearing who else is “authentically present” (38, 39), and how they are reacting to what is being discussed will have a positive effect on psychological safety for everyone involved, faculty included.
- Ensuring interactivity to enhance learning and build an online community: Educators utilize breakout rooms, polls, quizzes, or screen sharing to engage participants, identify possible learning gaps, create application opportunities, and provide external learning resources (40). Small group activities also foster engagement for those who are less comfortable speaking up in a large group.
- Feedback and evaluation: As with all other instructional settings, feedback must be bi-directional to promote a safer environment. Frequent quizzes are often used to help trainees gage their learning gains. Polls and evaluation tools that allow for anonymous responses help faculty understand the impact of their teaching. Consistent check-ins using multiple communication avenues ensure that no voices are lost in the virtual environment.

### Interprofessional education

In a psychologically safe team, interpersonal relationships are perceived as trusting and supportive, and everyone feels valued equally, regardless of individual roles. All participants must embrace and embody the core competencies and standards: values/ethics for interprofessional practice, roles/responsibilities, interprofessional communication, teams and teamwork (41). Psychological safety is critical to reducing patient care errors and enhancing learning outcome in the interprofessional teams (42). The following characteristics are critical (42):

- Open communications: Recognizing the difficulties of speaking up in a hierarchical system, the Agency for Healthcare Research and Quality developed TeamSTEPPS (43), a communication system that empowers everyone on the team to share observations and concerns. Other organizations created similar frameworks (44, 45). Training multi-professional teams on such communication strategies helps to empower everyone and creates an open communication culture.
- Diversity and inclusion: Healthcare teams include providers from different professions, each contributing unique skillsets. They may also come from diverse cultural and language backgrounds. Successful teams will bank on this diversity and create an inclusive environment whether it is for training or patient care.
- Well-defined team member roles and responsibilities: Understanding what is expected of oneself reduces uncertainty and thus increases psychological safety. While it is important to clarify everyone’s responsibilities upfront, roles can change over time. This can happen due to changes in training sites, a shift in team tasks, or additions or departures of team members. Each change requires a redefinition (and sometimes re-negotiation) of roles. Successful teams acknowledge that they are a dynamic social unit and that change can result in a threat to psychological safety that must be dealt with.
- Team leadership: Although psychological safety is easier obtainable in an egalitarian culture, leadership is also needed, even if it is rotating or episodic. Leaders will help the team maintain the focus on the common mission to accomplish learning tasks or provide optimal patient care. To effectively negotiate interpersonal conflicts and manage crisis situations leadership training must include the skills needed for developing a psychologically safe team culture.

## Discussion

Psychological safety is a critical factor for health professions education (HPE). These five types of HPE practices illustrate how the challenges and opportunities for psychological safety are embedded in training settings. Threats can be internal or external, and their causes are complex. Safety problems will have a ripple effect. If one person feels psychologically unsafe, it will undoubtedly change behaviors and thus affect others, overtly or covertly (46). All the featured training models emphasize the importance of reducing uncertainty because it breeds anxiety and anxiety results in psychologically unsafe conditions.

Cultural factors can significantly complicate the achievement of psychological safety (47). They can aggravate or mitigate personal and interpersonal challenges. What may seem safe for one person can be perceived as a threat by someone else who was brought up in a different social environment. To achieve “Safety for All,” educational programs must implement structures to enhance diversity awareness, facilitate acculturation, and celebrate belonging as a common good (48).

There are also systems-level problems. Healthcare is notoriously hierarchical, and open discussions about psychological safety can be challenging (49). However, raising awareness, prevention, and intervention strategies will be important for all stakeholders (50, 51). Accreditation requirements can help facilitate change. As previously mentioned, the ACGME recently included components of psychological safety as a core principle for residency programs (10). The new mandate calls for training programs to assess, implement and maintain a psychological safety culture. Programs are advised to regularly conduct anonymous surveys to assess psychological safety and invest in faculty and team development to create better learning and work environments.

Clark’s model provides a framework for assessing and implementing strategies to enhance psychological safety at individual, interpersonal and institutional levels (18). Relationship development and support for all involved are other common features that can help create a sense of security (49). Psychological safety is a critical element in every educational or healthcare enterprise. As complex as it is, educators need to be well-versed in identifying threats and utilizing well-established strategies to make programs psychologically safe (8, 51).

The strategies outlined in this article will benefit from further validation via rigorous experimental design to assess their effectiveness across various HPE settings. Key questions include how educators and learners perceive psychological safety in the different HPE settings, the development of tools to measure psychological safety in HPE, the influence of leadership type on psychological safety, and the relationship between psychological safety, stress reduction and its impact on burnout. Additionally, if educators and learners implement Clark’s model and the proposed strategies, it is essential to determine whether this leads to enhanced psychological safety and, consequently, improved learning outcomes. Moreover, to explore their applicability and effectiveness more broadly, these strategies could be implemented across diverse cultural contexts, allowing for an assessment of their efficacy in cross-cultural settings.

## Data Availability

The original contributions presented in the study are included in the article/supplementary material, further inquiries can be directed to the corresponding author/s.
